# Monitoring molecular response in adult T-cell leukemia by high-throughput sequencing analysis of HTLV-1 clonality

**DOI:** 10.1038/leu.2017.260

**Published:** 2017-09-05

**Authors:** M Artesi, A Marçais, K Durkin, N Rosewick, V Hahaut, F Suarez, A Trinquand, L Lhermitte, V Asnafi, V Avettand-Fenoel, A Burny, M Georges, O Hermine, A Van den Broeke

**Affiliations:** 1 Unit of Animal Genomics, GIGA-R, Université de Liège (ULg), Liège, Belgium; 2 Laboratory of Experimental Hematology, Institut Jules Bordet, Université Libre de Bruxelles (ULB), Brussels, Belgium; 3 Service d’Hématologie, Hôpital Universitaire Necker, Université René Descartes, Assistance Publique Hôpitaux de Paris, Paris, France; 4 Laboratoire d'Onco-hématologie, Institut Necker-Enfants Malades, INSERM U1151, Université Paris Descartes, Paris, France; 5 Laboratoire de Virologie, AP-HP, Hôpital Necker-Enfants Malades, Université Paris Descartes, Sorbonne Paris Cité, Paris, France; 6 Institut Imagine, INSERM U1163, CNRS ERL8654, Paris, France

Adult T-cell leukemia (ATL) is an aggressive CD4+ T-cell malignancy caused by the oncogenic retrovirus human T-cell leukemia virus-1 (HTLV-1).^1^ Although the cumulative incidence of ATL among HTLV-1-infected individuals is only 5%, it has an extremely poor prognosis and remains a major health concern in endemic regions.^2, 3^ Patients with an aggressive subtype have a median survival of 4–6 and 9–10 months for acute and lymphoma subtypes, respectively (Shimoyama classification^4^). Indolent forms (chronic and smoldering) have a more favorable median survival of 33 and 51 months. Treatment strategies for ATL mainly depend on clinical subtype, geographical localization and response to initial therapy.^5, 6^ In Japan, aggressive forms are treated by conventional chemotherapy combined or not with allogeneic stem cell transplantation,^7^ while in western countries the combination of zidovudine (AZT) with interferon (IFN) alpha is the standard first-line therapy for acute leukemic subtypes and chronic forms.^6^ Response to treatment and complete clinical remission are currently defined on the basis of cytomorphological consensus criteria that have not been revised over the 8 years since they were first described^8^ (Supplementary Methods). Given the extremely poor prognosis of ATL, the high rates of rapid relapse and the marked diversity in survival outcome after achieving hematological remission, there is an urgent need for new molecular tools that can reliably evaluate therapeutic response and better define remission.

The development of ATL is associated with the emergence of a dominant clone uniquely identified by the proviral integration site within the host genome, with an underlying polyclonal population of infected cells of varying abundance.^9, 10^ In the majority of ATL cases examined to date, the presumed malignant clone carries a single proviral integration.^9, 10, 11^ In this study, we explored the benefit of an optimized high-throughput sequencing (HTS) clonality method as a quantitative molecular approach to monitor the malignant clone identified at diagnosis and better evaluate therapeutic response.^10, 12^ The method enables the genome-wide mapping of HTLV-1 integration sites and the simultaneous quantification of the abundance of the corresponding clones. It includes several critical modifications that overcome the limitations of previously reported protocols,^13, 14^ increasing sensitivity, facilitating multiplexing, and significantly reducing both the cost and hands-on time (Supplementary Methods). As a proof-of-concept, we analyzed retrospective longitudinal samples of five ATL patients diagnosed with a leukemic subtype who all achieved complete hematological remission upon induction therapy. Although all five patients eventually relapsed, the duration of hematological remission and the clinical course were variable between patients (Supplementary Table 1). Two patients achieved a protracted clinical remission of 5.8 and 2.4 years (ATL11 and ATL60; Figures 1a and b), while three patients relapsed after a significantly shorter remission of 4.3, 5.3 and 3.7 months for ATL7, ATL14 and ATL100, respectively (Table 1, Figures 1c–e).

For each ATL patient, we analyzed the clonal architecture (i) at diagnosis, (ii) at relapse and (iii) at intermediate time points that consisted of either a single (CR1; ATL7 and ATL100) or multiple (CR1, CR2, CR3; ATL11, ATL14 and ATL60) longitudinal samples collected at hematological remission. PVL (proviral copies per 100 peripheral blood mononuclear cells), T-cell receptor (TCR)-γ rearrangement and blood immuno-phenotypes were also recorded (Table 1). HTS mapping of HTLV-1 integration sites at diagnosis revealed a single dominant integration site that constituted 92.75 to 99.86% (mean 95.9%) of proviral genomes in four ATL cases (ATL7, ATL11, ATL14 and ATL100). In the remaining tumor (ATL60), there was evidence of four dominant proviruses present at the same frequency in a single malignant clone, consistent with the observation of a single TCR-γ rearrangement (total relative abundance 99.05%). All patients were treated and achieved complete clinical remission (Supplementary Table 1 and Supplementary Methods). For 2/5 patients, molecular analysis revealed that the predominant HTLV-1 insertion site of the presumed malignant clone fell from 97.32 to 1.87% (ATL11) and from 99.05 to 2.15% (ATL60) following treatment. In both patients, the clone frequency distribution of HTLV-1-infected cells at clinical remission was composed of multiple low abundance clones, of which the unique presumed malignant integration site contributed to <3% of proviral genomes (Figures 1a and b, CR1). One patient (ATL11) remained in clinical and molecular remission for 5 years and 11 months with no significant change in clone frequency and modest fluctuations in PVL (CR2 and CR3; Figure 1a), while the second patient (ATL60) showed a gradual yet moderate recurrence of the malignant clone over the 2 year and 4 month period of complete hematological remission with no increase in PVL (9.25 and 36.95%, CR2 and CR3, Figure 1b). Clonality analysis of ATL60 at relapse revealed the full recurrence of the predominant integration sites detected at diagnosis with clonal abundance of 99.46%. ATL11 relapsed with a lymphoma subtype and a different clone (89.3% abundance), consistent with previous reports of clonal transition during the clinical course of ATL.^15^ The dominant integration site was supported by 3′ long terminal repeat (LTR)–host junctions yet 5′LTR-dependent reads were not retrieved in the sequencing output, strongly suggesting that the new malignant clone that emerged at relapse carried a 5′LTR-deleted provirus. We verified that this was the case by applying the long-range *Oxford Nanopore* sequencing technology to characterize the provirus and its genomic boundaries in the lymphoma that developed at relapse (Supplementary Figure 1). This demonstrated the ability of our 5′/3′ dual HTS approach in faithfully identifying 5′LTR-deleted type 2-defective proviruses that have been observed in one-third of ATLs and have prognostic value.^9, 10, 11^ Clonality analysis of the remaining 3/5 patients after induction therapy revealed that, contrary to ATL11 and ATL60, the relative abundance of the malignant clone identified at diagnosis remained dominant at clinical remission (73.4, 43.24 and 55.43% at CR1; 92.75, 99.86 and 94.95% at diagnosis for ATL7, ATL14 and ATL100, respectively) while the clinical response criteria were consistent with complete hematological remission and the PVLs had decreased 1.7–>1000-fold (Table 1 and Figures 1c–e). These patients relapsed after 4.3, 5.3 and 3.7 months, respectively, with the dominant malignant clone >86% (86.20, 88.1, and 99.12%, respectively). Thus the molecular follow-up of these patients revealed refractoriness to first-line therapy at time points where clinical response criteria indicated complete hematological remission. Interestingly, while lymphocyte counts and blood smears remained unremarkable during complete remission in ATL14 (Table 1), longitudinal HTS revealed molecular recurrence of the malignant clone at these time points (abundance of 43.24–70.84%, CR1 and CR3). This patient relapsed with strong lymph node involvement (LN++), yet blood parameters remained in the normal range. This demonstrates that HTS analysis of the blood of a patient in complete hematological remission can predict relapse at distant sites.

We also analyzed TCR-γ rearrangement and flow cytometry (FCM) immuno-phenotype profiles for all samples (Figure 1 and Table 1). These assays appeared to be better indicators of recurrence than the standard hematological response criteria at certain time points, yet both had their limitations. With regards to TCR-γ, HTS clonality was a superior predictor of refractoriness to induction therapy (Figure 1, ATL100 CR1, ATL14 CR1 and CR2). It also enabled the quantitative assessment of recurrence after a short period of molecular remission (Figure 1, ATL60) and showed increased sensitivity (Figure 1d, CR1, 0.007% absolute abundance versus ~1% sensitivity for TCR-γ assay). FCM revealed an abnormal lymphocyte population that expressed CD4^+^/CD25^+^/CD7^−^/CD3^dim^ in 4/5 ATLs; however, the interpretation of FCM profiles for accurate detection of residual disease based on CD3^dim^ status remained problematic given the overlap of CD3 levels with the distribution observed in healthy individuals. Furthermore, not all ATL cases show CD3^dim^ phenotypes (Table 1, ATL100: CD3^high^), demonstrating the limitations of FCM as a marker of molecular response. Finally, RNA-seq of ATLs at diagnosis and relapse revealed the consistent production of HBZ antisense transcripts while 5′LTR-dependent sense transcription—including Tax—was undetectable, consistent with previously published studies of our group and others.^10, 11^ As expected, we did not observe significant differences in HBZ levels between diagnosis and relapse (*P*=0.3946, Table 1, Supplementary Table 2). Altogether, these observations support the conclusion that the HTS clonality method is superior to any other assay tested thus far.

In summary, this pilot study shows that molecular knowledge with regards to HTLV-1 clonal architecture and follow-up of the dominant leukemic clone over time enable a more reliable definition of remission and a better estimate of molecular response in ATL patients. HTS can reveal ATLs refractory to first-line therapy or detect molecular relapse in patients that achieve hematological remission. Additionally, the method can identify 5′LTR-variants, clone switch upon progression and is superior to any conventional method available thus far. The optimized protocol overcomes previous limitations in terms of sensitivity, cost and hands-on time, facilitating implementation in the clinic. Our data support its incorporation in routine follow-up, providing clinicians with a tool to rapidly identify patients who do not benefit from standard therapy and should be enrolled in alternative or novel upfront strategies. It is possible to integrate this approach into next-generation sequencing-based mutation profiling schemes that are increasingly used for monitoring patients with hematological malignancies. As such, although small scale, the study by itself provides a strong proof-of-concept to build on and establishes the rationale for further clinical evaluation. Efforts of the HTLV-1 community will be needed to define how HTS clonality data should be reported for clinical utilization and delineate the optimal time/interval for assessing molecular response in standardized care.

Altogether, our observations highlight the great molecular heterogeneity within patients who achieve complete clinical and hematological remission, underlining the need for revisiting response criteria for ATL. We propose this HTS approach as a method to detect minimal residual disease, estimate graft-versus-ATL effect after allogeneic stem cell transplantation and evaluate clinical trials that remain critical to improving outcomes.

## Figures and Tables

**Figure 1:**
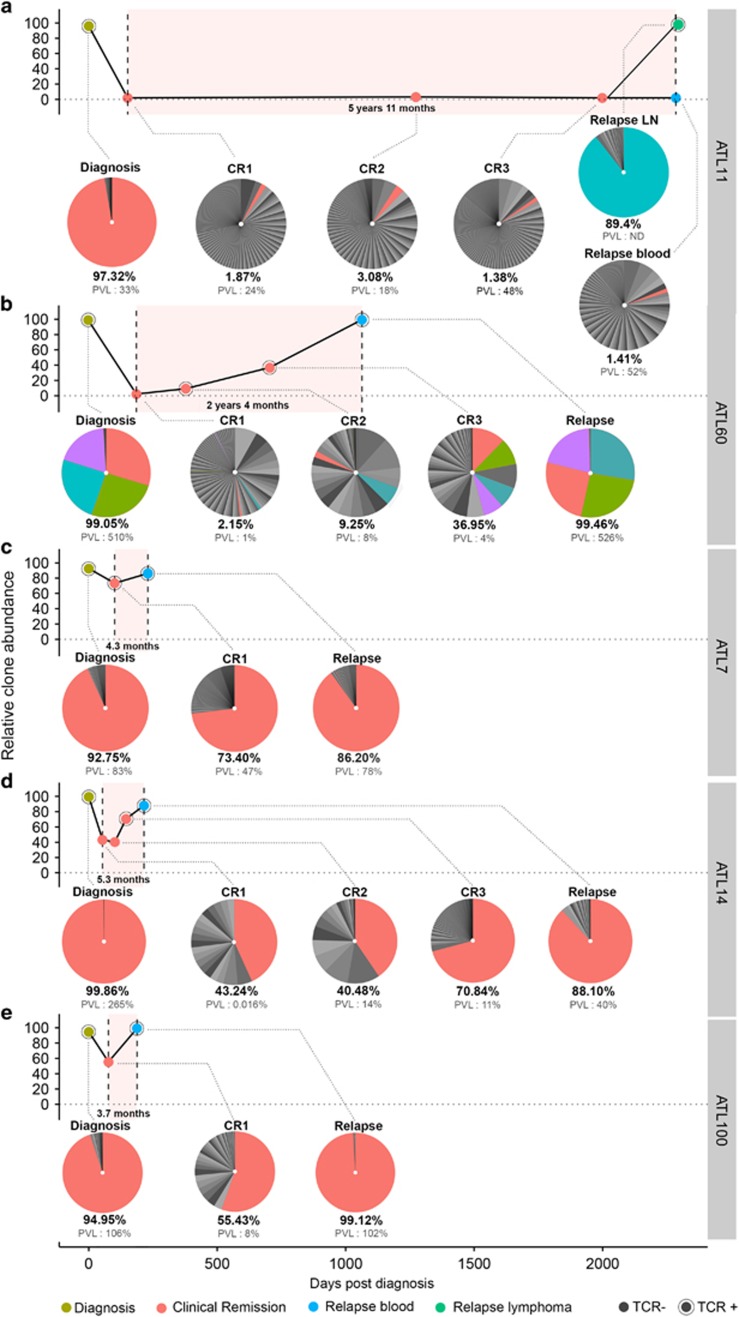
Longitudinal monitoring of the dominant malignant clone and clone frequency distribution in five ATL patients. (**a**–**e**) Evolution of the abundance of the dominant clone relative to all infected cells is represented by longitudinal charts with colored dots corresponding to each time point (diagnosis, relapse, complete remission CR1, CR2 and CR3). Pink area with red dots indicates the period of complete clinical remission (Supplementary Table 1). Samples with a clonally rearranged TCR-γ gene have dots marked with a circle (TCR^+^). Clone frequency distribution is illustrated by pie charts, each slice representing an independent integration site and its corresponding clonal abundance. The dominant clone (relative abundance per 100 proviral copies, indicated below the pie chart) is depicted in red except for ATL11-Relapse-LN (turquoise, clone switch) and ATL60 (four equally frequent proviruses in a single malignant clone, single TCR-γ rearrangement, Table 1). The remaining underlying clones are shown in gray. PVL: proviral load (tax copies per 100 peripheral blood mononuclear cells (PBMCs)). Absolute abundance (percentage of HTLV-1 insertion sites in PBMCs) was calculated from PVL and the clone’s relative abundance. Absolute abundance of malignant integration sites at CR ATL14-CR1<0.007% (**d**, PVL: 0.016%, relative abundance within HTLV-1-infected PBMCs, 43%).

**Table 1:** Patients’ clinical and molecular characteristics

*Patient*	*Status*	*ALC (G/l)*	*Blood smear*	*ATL localization*	*Calcemia (mmol/l)*	*LDH*	*TCR*	*FCM %*	*CD4/CD8%*	*HBZ*	*PVL %*
ATL7	D	43.9	>5%	Blood, LN	2.38	4*N*	+	>80	2.43/3.28	×	83
	CR1	1.3	—		2.21	1.5*N*	+	25	24.1/12.7		47
	R	3.5	>5%	Blood, CNS	2.26	7*N*	+	50	11.7/8.1	×	78
ATL11	D	1.3	>5%	Blood	2.27	<1*N*	+	40	37.7/9.5	×	33
	CR1	2.1	<5%		2.31	1.5*N*	−	4	14.50/44.5		24
	CR2	1.4	—		2.32	<1*N*	−	<5	23.3/25.6		18
	CR3	1.6	—		ND	<1*N*	−	4	34.2/16.1		48
	R	1.8	<5%	LN	2.49	2*N*	−				
				Lymphoma			+^a^	6	27.1/17.6		52
ATL14	D	16.4	>5%	Blood, LN	2.17	17*N*	+	85	2.5/2.0	×	265
	CR1	0.2	—		2.3	6*N*	−	0.20	8.0/11.0		0.016
	CR2	0.6	—		2.4	1.5*N*	−	6	12.3/27.3		14
	CR3	1.2	—		2.46	1.5*N*	+	10	15.6/21.8		11
	R	1.9	>5%	Blood, LN++	2.5	ND	+	14	18.2/19.6	×	40
ATL60	D	7.8	>5%	Blood, LN	2.97	5*N*	+	>50	6.4/21.3	×	510
	CR1	1.3	—		2.36	2*N*	−	<1	13.4/28.7		1
	CR2	2.8	—		2.31	1.5*N*	+	4	18.4/53.7		8
	CR3	2.6	—		Normal	1.5*N*	+	6	16.6/56.3		4
	R	47.2	>5%	Blood	3.7	6*N*	+	78	2.7/11.5	×	526
ATL100	D	8.0	>5%	Blood, liver	Normal	6*N*	+	>70^b^	3.8/3.7		106
	CR1	3.2	—		Normal	1.5*N*	−	3^b^	15.7/17.3		8
	R	12.4	>5%	Blood, liver	Normal	5*N*	+	>95^b^	1.3/1.3	×	102

Abbreviations: ALC, absolute lymphocyte count; ATL11 D: corresponds to the earliest available sample from this patient (non-responder clinical status after chemotherapy, prior to AZT-IFN alpha-As_2_0_3_ treatment); Blood smear, the presence of flower cells (%); CD4/CD8, percentage CD4^+^ and CD8^+^ lymphocytes determined by FCM; CR, complete remission; D, diagnosis; FCM, percentage CD4^+^*/*CD25^+^/HLADR^+^/CD7^−^/CD3^*dim*^ lymphocytes in PBMCs determined by flow cytometry immuno-phenotyping; HBZ, indicates ATLs at diagnosis and relapse for which HBZ transcripts were quantified by RNA-seq; there was no significant difference in the HBZ expression levels between D and R (*P=*0.3946, global analysis combining this data set and a previously published ATL data set,^10^ Supplementary Table 2 and Supplementary Methods); LHD, lactate dehydrogenase; *N*, normal values; ND, not done or not available; PVL, proviral load in copies of tax per 100 PBMCs; R, relapse; TCR, clonal TCR-γ rearrangement in the blood.

a TCR-γ rearrangement in lymphoma, distinct clone.

b ATL100 characterized by CD4^+^/CD25^+^/HLA DR^+^/CD7^−^/CD3^*high*^ cell population.
