# Supplementary material for: Monitoring molecular response in adult T-cell leukemia by high-throughput sequencing analysis of HTLV-1 clonality
Source: Leukemia. 2017 Sep 5;31(11):2532–5. doi: 10.1038/leu.2017.260 (PMC5668493; doi:10.1038/leu.2017.260)
Supplement: Supplementary Information [file leu2017260x1.pdf]

Supplementary Information

Monitoring molecular response in adult T-cell leukemia by high-throughput sequencing analysis of HTLV-1 clonality

Maria Artesi, Ambroise Marçais, Keith Durkin, Nicolas Rosewick, Vincent Hahaut, Felipe Suarez, Amélie Trinquand, Ludovic Lhermitte, Vahid Asnafi, Véronique Avettand-Fenoel, Arsène Burny, Michel Georges, Olivier Hermine and Anne Van den Broeke

The Supplementary information file contains Supplementary Methods, Supplementary Figures (1), Supplementary Tables (2), and Supplementary References.

Supplementary Methods

Study design and Patients

The study was conducted on 22 retrospective longitudinal samples of five ATL patients diagnosed with an aggressive leukemic subtype, treated at the Necker Hospital (Paris) between 2008 and 2016, and for which serial samples during clinical remission and at relapse had been archived (Table 1 and Supplementary Table 1). The study was approved by the ethics committee CPP Ile-de-France II and all patients gave written informed consent if not deceased. Diagnosis of ATL was based on clinical parameters, the presence of atypical lymphocytes in blood smears and the presence of HTLV-1-specific antibodies in serum. Patients were classified into lymphoma, acute, chronic and smoldering subtypes according to the Shimoyama classification.[1] Induction therapy consisted of either a CHOP-based chemotherapy regimen or zidovudine (AZT) + interferon (IFN)-alpha combination therapy[2]. As_20_3 was used as consolidation therapy in two cases. Patients' characteristics are summarized in Supplementary Table 1. Complete hematological remission was defined by morphological and cytological criteria according to the recommendations published in 2009[3] i.e. the normalization of CBC, the presence of < 5 % abnormal lymphocytes in the blood and the absence of measurable tumors for > 4 weeks. Patients' CBC, absolute lymphocyte counts (ALC), blood smears, clinical data and biological parameters determined by European standard protocols in the accredited diagnostic laboratory of Onco-hematology were retrospectively obtained from electronic medical records at the Necker Hospital (Table 1). DNA used in this study was isolated using Qiagen AllPrep-DNA kit.

HTS mapping of HTLV-1 integration sites and measure of clonal abundance

An improved HTS-based method was utilized to simultaneously map and quantify the abundance of HTLV-1 integration sites in genomic DNA isolated from the patients'

longitudinal PBMC samples.[4,5] In addition to the random tags described in the Tag-NGS protocol developed by Watanabe and colleagues[6], our optimized method includes several critical modifications in library preparation and data analysis, overcoming some of the limitations of previously published protocols.[6–9] Briefly, the dynamic range of the technique was increased by assaying both the 5'LTR and 3'LTR (5'/3' dual method), allowing better determination of clone abundance and providing critical information on the occurrence of 5'-deletions in the provirus. An extension step with Biotin-11-dUTP simultaneously end-repairs and facilitates streptavidin-based enrichment of LTR-positive fragments, increasing the sensitivity of the assay, followed by limited PCR to reduce PCR duplicates. Off-the-shelf Illumina primers replaced custom sequencing primers for the addition of adapters and indexes, simplifying library multiplexing and reducing both the cost and hands-on time to the point where the protocol can be applied to a clinical setting. Libraries were assembled and 150-bp paired-end sequencing reads were acquired on an Illumina MiSeq instrument (mean number of raw reads: 373,400, range: 28,930-977,000). Reads that supported either 5' or 3'LTR-host junctions were retained. The number of unique HTLV-1 integration sites and the abundance of the corresponding clones were determined as described. The clonal architecture of ATL samples can be typically determined from 50,000 to 200,000 raw reads (0.25% to 1% of MiSeq output), decreasing the overall cost per sample 10- to 40-fold. A clone's relative abundance corresponds to the percentage of that particular clone within the population of provirus-positive cells. Absolute clone abundance represents the percentage of PBMCs (whether infected or not) that carry the corresponding insertion site, and is calculated from the clone's relative abundance and the sample PVL. For complete methodologic details, see Rosewick *et al.*[4] and Percher *et al.*[5]

Long-range Oxford Nanopore sequencing of clinical samples

HTLV-1 proviruses and their respective 5’ and 3’flanking genomic sequences were PCR-amplified using LongAmp® Taq DNA Polymerase (NEB). ATL14 (presumed full-length provirus, 5’LTR-host and 3’LTR-host junctions detected by HTS clonality): primers Forward-5-TGGGGCGACATCTGAAGAAA-3 & Reverse-5-TGCAGGGTTGGAGTTTCAGA-3 produced a 750-bp (wild-type chromosome) and ~9000 bp band including the provirus. ATL11-R-LN (lymph node, relapse): primers Forward-5-CCTTAATCACGCTCTGGTGC-3 & Reverse-5-GCGGACTTGGGCCTTATCAT-3 produced a 460-bp band (wild-type chromosome) and a ~4000 bp band including presumed 5’LTR-deleted type-2 defective provirus. Libraries prepared from gel-purified PCR products (Ligation Sequencing kit 2D-R9.4) were sequenced with a SpotON Flow Cell Mk-I-R9.4 (Oxford Nanopore Technologies). FASTQ sequences were extracted using Poretools[11] and mapped via BWA-MEM[12] to custom genomes with provirus integrated into the appropriate sites.

T-cell receptor (TCR) gene rearrangement

TCR-gamma (γ) gene rearrangement was assessed using the established Euroclonality (Biomed-2) protocol that was integrated in the routine assessment of hematological malignancies at the Necker hospital.[13]

Flow Cytometry (FCM) Immuno-phenotyping of blood cells

The presence of abnormal lymphocyte populations that express CD4 (RPA-T4), CD25 (2A3), HLA-DR (L243), $CD3^{dim}$ (UCHT1) and $CD7^{absent}$ (124-1D1), and the percentage $CD4^+$ and $CD8^+$ (SK1) cells in the blood of ATL patients was examined by FCM according to Euroflow standardization protocols (antibodies were from BD Biosciences except for 124-1D1 that was

purchased from eBioscience). FCM analysis has been integrated in the routine assessment of hematological malignancies at the Necker hospital.

Quantification of HBZ expression levels by RNA-seq

Total RNA was extracted using the AllPrep DNA/RNA kit (Qiagen). Strand-specific ribosomal RNA depleted RNA-seq libraries prepared using the Illumina TruSeq Total RNA stranded kit were sequenced on a Nextseq500 instrument (Illumina, 2 x 75 bp paired-end reads) and HTLV-1 transcripts were quantified as previously described[4]. HBZ expression levels were normalized to both sequencing depth and proviral load. *p*-values reflecting differences in HBZ levels between ATLs at diagnosis and relapse were determined by combining RNA-seq data of previously-sequenced ATLs[4] and the RNA-seq data of this study (Table 1, Supplementary Table 2) using a two-tailed Mann-Whitney U-test.

HTLV-1 proviral load

Proviral load (PVL), the number of proviral copies per 100 PBMCs was monitored by real-time PCR as previously described.[4]

Data availability

HTLV-1 genomic integration information that supports the findings of this study have been deposited in the Retroviral Integration Database (RID)[10]. All other relevant data are available within the article and its Supplementary Information files or from the corresponding author upon request.

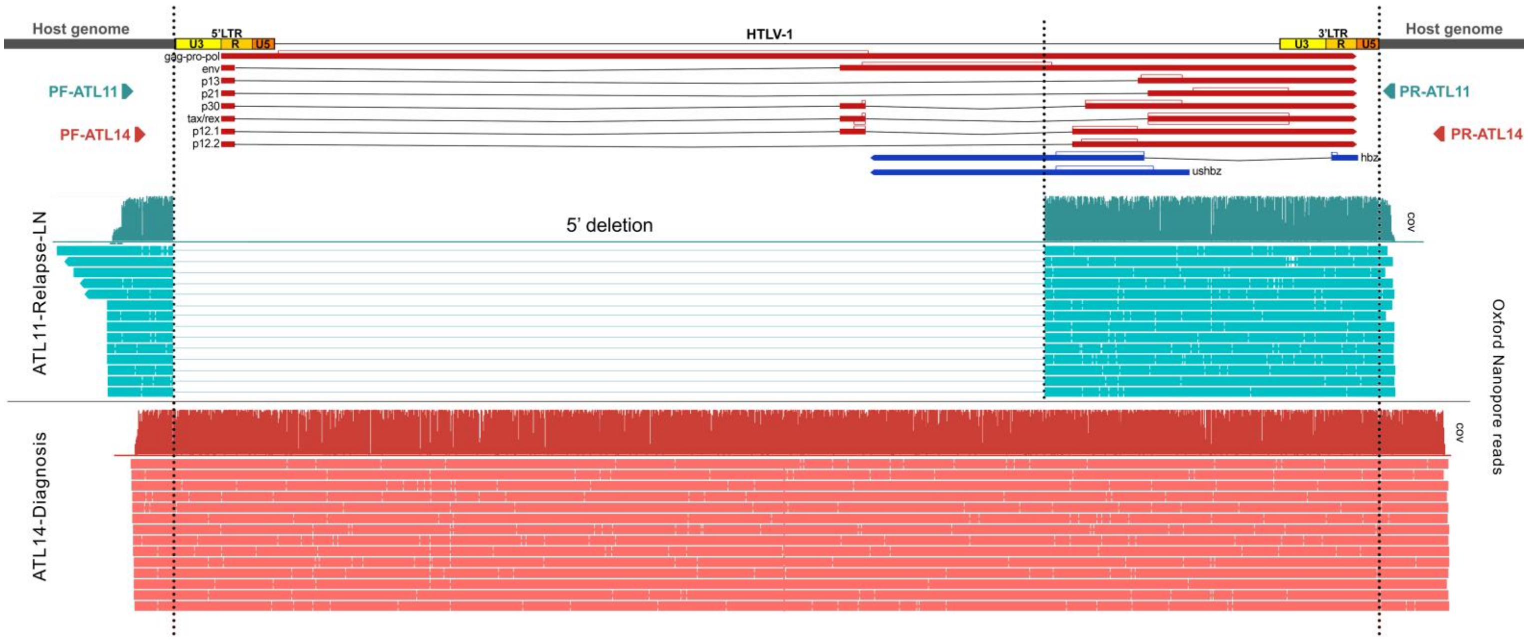

Supplementary Figure 1. Long-range Oxford Nanopore sequencing validates the capacity of 5'/3' dual HTS clonality method in revealing HTLV-1 type-2 defective proviruses

Coverage and individual reads generated by Oxford Nanopore sequencing of PCR products obtained with primer pairs located up- (PF) and downstream (PR) of the predominant HTLV-1 integration site in ATL11-Relapse-LN (top) or ATL14 Diagnosis (bottom) visualized in IGV[14] and mapped to custom genomes that have the provirus integrated into chr 1: 20,516,805 (ATL11-Relapse-LN) or chr 18: 45,011,572 (ATL14 D) in the human genome. Integration sites were identified by HTS clonality mapping. Reads spanning both human and proviral genome were on average 2,143 bp in length for ATL11-R-LN (range 41 bp to 9,155 bp) and 3,255 bp for ATL14 (range: 73 bp to 9,562 bp). ATL11-relapse-LN coverage uncovers a large 5' deletion of 6,529 bp in the proviral genome (includes 5'LTR), consistent with the absence of 5'LTR-host reads observed with the HTS clonality method while 3'LTR-host junctions were detected. Long reads validate 5'/3' dual HTS method for identifying type-2 defective HTLV-1 proviruses in ATL. HTLV-1 proviral genome and transcripts shown on top. PF: primer forward, PR: primer reverse, cov: coverage.

Supplementary Table 1. Patients' characteristics

Patient	Sex/Age (years)	Ethnic origin	Subtype at diagnosis	Treatment	CR (months)	Time between diagnosis and relapse (months)
**ATL7**	F/59	Caribbean	Acute	CHOP regimen	4.3	7.6
**ATL11**	M/27	French Guyana	Unfavorable chronic	LSG 15 & AZT-IFN alpha AS_2O_3	70.7	75.7*
**ATL14**	F/35	Haiti	Acute	CHOP regimen (induction) & AZT-IFN alpha AS_2O_3 (consolidation)	5.3	7.2
**ATL60**	M/42	Africa	Acute	CHOP like regimen	28	34
**ATL100**	F/55	Africa	Acute	AZT-IFN alpha	3.7	6.3

F: female, M: male, Subtype defined according to Shimoyama classification[1], CHOP: Cyclophosphamide, Adriamycin, Vincristine, Prednisone, LSG 15: VCAP-AMP-VEPC, IFN alpha: Interferon alpha, AS_2O_3: Arsenic trioxide, CR: complete remission. AZT-IFN alpha treatment consisted of 4 - 6 week induction therapy followed by maintenance antiviral therapy[15,16]. *ATL11 diagnosis corresponds to the earliest available sample from this patient (non-responder clinical status after chemotherapy, prior to AZT-IFN alpha-AS_2O_3 treatment).

Supplementary Table 2. HBZ expression levels at diagnosis and relapse

Patient	Status	PVL %	HBZ (TPM)	HBZ (TPM/PVL)
**ATL7**	D	83	1.447	1.743
	R	78	1.055	1.352
**ATL11**	D	33	0.840	1.867
	R	6	NA	NA
**ATL14**	D	265	1.395	0.526
	R	40	0.370	0.926
**ATL60**	D	510	6.115	1.199
	R	526	1.525	0.289
**ATL100**	D	106	NA	NA
	R	102	2.920	2.863

HTLV-1 HBZ antisense transcripts in ATLs were analysed by stranded RNA-seq. HBZ TPM: transcripts per million normalized on sequencing depth, TPM/PVL: TPM normalized on proviral load (PVL). Normalized HBZ expression levels of this dataset (8 ATLs, D: diagnosis, R: relapse) were combined with the levels observed in a previously-published dataset[4] (19 ATLs with known PVL), and HBZ expression at diagnosis and relapse were compared using a two-tailed Mann Whitney U-test. $p = 0.2828$ (TPM), $p = 0.3946$ (TPM/PVL)

Supplementary References

1 Shimoyama M. Diagnostic criteria and classification of clinical subtypes of adult T-cell leukaemia-lymphoma. A report from the Lymphoma Study Group (1984-87). Br. J. Haematol. 1991; **79**: 428–437.

2 Bazarbachi A, Suarez F, Fields P, Hermine O. How I treat adult T-cell leukemia/lymphoma. Blood. 2011; **118**: 1736–1745.

3 Tsukasaki K, Hermine O, Bazarbachi A, Ratner L, Ramos JC, Harrington W *et al.* Definition, prognostic factors, treatment, and response criteria of adult T-cell leukemia-lymphoma: a proposal from an international consensus meeting. *J Clin Oncol* 2009; **27**: 453–9.

4 Rosewick N, Durkin K, Artesi M, Marçais A, Hahaut V, Griebel P *et al.* Cis-perturbation of cancer drivers by the HTLV-1/BLV proviruses is an early determinant of leukemogenesis. *Nat Commun* 2017; **8**: 15264.

5 Percher F, Curis C, Pérès E, Artesi M, Rosewick N, Jeannin P *et al.* HTLV-1-induced leukotriene B4 secretion by T cells promotes T cell recruitment and virus propagation. *Nat Commun* 2017; **8**: 15890.

6 Firouzi S, López Y, Suzuki Y, Nakai K, Sugano S, Yamochi T *et al.* Development and validation of a new high-throughput method to investigate the clonality of HTLV-1-infected cells based on provirus integration sites. Genome Med. 2014; **6**: 46.

7 Gillet N, Malani N, Melamed A, Gormley N, Carter R, Bentley D *et al.* The host genomic environment of the provirus determines the abundance of HTLV-1-infected T-cell clones. Blood. 2011; **117**: 3113–3122.

8 Cook LB, Melamed A, Niederer H, Valganon M, Laydon D, Foroni L *et al.* The role of HTLV-1 clonality, proviral structure, and genomic integration site in adult T-cell leukemia/lymphoma. Blood. 2014; **123**: 3925–3931.

9 Aoki S, Firouzi S, López Y, Yamochi T, Nakano K, Uchimaru K *et al.* Transition of adult T-cell leukemia/lymphoma clones during clinical progression. *Int J Hematol* 2016; **104**: 330–337.

10 Shao W, Shan J, Kearney MF, Wu X, Maldarelli F, Mellors JW *et al.* Retrovirus Integration Database (RID): a public database for retroviral insertion sites into host genomes. *Retrovirology* 2016; **13**: 47.

11 Loman NJ, Quinlan AR. Poretools: a toolkit for analyzing nanopore sequence data. *Bioinformatics* 2014; **30**: 3399–3401.

12 Li H. Aligning sequence reads, clone sequences and assembly contigs with BWA-MEM. 2013.http://arxiv.org/abs/1303.3997 (accessed 22 Feb2017).

13 Van Dongen JJM, Langerak AW, Brüggemann M, Evans PAS, Hummel M, Lavender FL *et al.* Design and standardization of PCR primers and protocols for detection of

clonal immunoglobulin and T-cell receptor gene recombinations in suspect lymphoproliferations: Report of the BIOMED-2 Concerted Action BMH4-CT98-3936. *Leukemia* 2003; **17**: 2257–2317.

14 Thorvaldsdottir H, Robinson JT, Mesirov JP, Thorvaldsdóttir H, Robinson JT, Mesirov JP. Integrative Genomics Viewer (IGV): high-performance genomics data visualization and exploration. Brief. Bioinform. 2013; **14**: 178–192.

15 Hermine O, Allard I, Lévy V, Arnulf B, Gessain A, Bazarbachi A. A prospective phase II clinical trial with the use of zidovudine and interferon-alpha in the acute and lymphoma forms of adult T-cell leukemia/lymphoma. Hematol. J. 2002; **3**: 276–282.

16 Bazarbachi A, Plumelle Y, Carlos Ramos J, Tortevoye P, Otrock Z, Taylor G *et al.* Meta-analysis on the use of zidovudine and interferon-alfa in adult T-cell leukemia/lymphoma showing improved survival in the leukemic subtypes. *J Clin Oncol* 2010; **28**: 4177–83.
